# Science under the lamppost

**DOI:** 10.1186/1478-811X-9-21

**Published:** 2011-09-22

**Authors:** Stephan M Feller

**Affiliations:** 1 Weatherall Institute of Molecular Medicine, John Radcliffe Hospital, Oxford University, Oxford OX3 9DS, UK

## Science under the lamppost

In these times of financial folly, there exists the serious threat that many scientists will soon be forced to work like the man who searches for his lost key under the lamppost because that is the only place where he can easily see.

As we have moved from the age of manic predator capitalism into the embarrassing age of 'pampers capitalism' (i.e. the common man has to clean up the mess created by irresponsible bankers and is even expected to pay for the nappies to catch the future fallout that is very likely to occur), the pressure grows immensely to fund only science that appears to have such an obvious and quick payoff for society that any dimwit can spot without a problem.

Short-sighted science administrators/managers who are primarily eager to keep a low profile and politicians who are largely focussed on being re-elected love this kind of science, especially if it is linked to fancy gadgets.

This coincides nicely with the flourishing of high-throughput (HTP) platform technologies that produce monstrous amounts of data. So far physics has been the science to churn out terabytes of data per day, but soon biomedicine, for example large nucleic acid sequencing projects and high throughput proteomics, will surpass the physicists with ease in their ability to generate vast piles of data that no one understands. It will be all but impossible to effectively extract most information from these piles, in part because the conceptual frameworks for this are still missing.

Cell signalling networks are a case in point. Even the most complex network charts or depictions of signalling hub collections and their connections still resemble the drawings primary school children in their simplicity. An overarching concept of how large signalling networks really function *in vivo *does not exist [1], in part because the molecular architectures of large signalling complexes and their *in vivo *connections remain practically unknown. So far, the fact that signalling occurs in an extremely crowded intracellular environment, and therefore must be highly coordinated not only in time but also in 'nanospace', is only on the agenda of a relatively small number of scientists. This is not surprising, because this research area still needs to develop many new tools to move forward effectively. Thus it represents an area at some distance from the lamppost and finds it more difficult to attract funding in these times of scarce research budgets. Nevertheless, we should not accept this neglect without a fight, since we will need to progress in this realm, if we want to develop a deeper understanding of the biology of signalling networks, genomes etc.

What can we, the scientific community, do about the threat of being forced to perform more and more boring searches under the lamppost?

For one, a 'new honesty' is needed. Scientists with inflated egos need to stop pretending that most amazing discoveries arise from the sheer brilliance of their great minds in combination with huge machines. We must make the common man understand that science is often a fairly erratic process, sometimes complicated by the all too human shortcomings of investigators [2] and that real breakthroughs can never be reliably predicted in advance. Some great concepts or findings may sit around for many decades until their true value is recognised [3].

In other words, we will need to become more humble and frank with regard to our actual ability to tackle big biological questions systematically, even in the 21^st ^century.

Second, we will not only need more bioinformaticians to help us sift through massive data piles; we must also encourage 'theoretical biologists' to come out of their closets and to boldly stick their necks out by presenting daring new concepts of how complex biological systems may function.

In addition, we must allow more 'off the wall' questions and dare to think more out of our little boxes...'Is there room for the 'butterfly effect' (chaos theory) in cell signalling?' If not: 'How do cells escape?' (redundancy, robustness); 'What do fractals tell us about cancer cell signalling?' (see also [4]) etc.pp.

Curiously, slowing down the pace of the large and costly HTP-gadgets that start to produce data at hyper-speed, due to the imminent lack of funds, might possibly also have its benefits; we may find that we have just a bit more time to think carefully about how best to conduct experiments and how to properly evaluate the quality and meanings of their results.

Without a doubt, we live in interesting times, but this could turn out to be a blessing as much as it might become a curse.
